# An integrated approach to the development of a Bayesian response-adaptive dose-finding study using sas and winbugs

**DOI:** 10.1186/1745-6215-14-S1-O74

**Published:** 2013-11-29

**Authors:** Christian Holm Hansen, Christopher Weir, Pamela Warner, Hilary Critchley

**Affiliations:** 1 University of Edinburgh, Edinburgh, UK

## 

Heavy Menstrual Bleeding (HMB) is common and existing treatments often ineffective. The MRC funded DexFEM trial aims to investigate whether oral dexamethasone (Dex; a glucocorticoid) reduces HMB.

Our integrated approach to the development of a Bayesian response-adaptive dose-finding study uses SAS for data handling, generating scripts and executing analysis in WinBUGS. The clinical aims are twofold: (i) to determine if Dex is efficacious in reducing HMB compared with placebo and, if so, (ii) to identify the optimal dose for further phase III study. Patients are randomised to placebo or one of several active Dex doses. Randomisation probabilities change as outcome data accumulate from patients already recruited. This design ensures that maximum information is gathered on the dose-response curve, and also that fewer women are randomised to inferior doses. Development of an adaptive design cannot invoke standard frequentist concepts of power. We estimate the dose-response curve in a Bayesian Normal Dynamic Linear Model.

Several competing design options are considered including: the number of doses; the criterion for adapting the randomisation; the length of the run-in before adaptation commences; the frequency with which randomisation probabilities are adapted. Through extensive simulation work we assess the performance of candidate designs under a variety of plausible scenarios for dose-response curve shape and outcome measure variance, and decide on a final design. Adaptive designs offer flexibility and efficiency and are likely to be used more in the future. Our integrated approach is a practical, powerful tool for the development of such designs.

